# Scirrhus of the Pancreas

**Published:** 1878-11

**Authors:** J. F. Waid


BUFFALO

### Medical and Surgical Journal.

VOL. XVIII. NOVEMBER, 1878. NO. 4.

### Original Communications.

:o:

*ART. I.—Scirrhus of the Pancreas—Cases in Practice.*—By J. F. WAID, M. D.

Recognizing the fact that any disease of the pancreas is difficult of diagnosis, I report the following, thinking they may be of interest to others as they have been to me:

CASE 1.—Mr. M——, aged 48, married, by occupation a lumberman. Came to me in the summer of 1870; complained of pain in stomach, pain aggravated if fatty food was taken. Prescribed counter-irritation over epigastrium, internally bismuth and tonics; with these he retired. Again in 1871 he returned, the pain more acute; seemed neuralgic in character. At times entirely relieved, then paroxysms of very acute pain. Diagnosed neuralgia of stomach. Prof. Gross being consulted made the same diagnosis. Treatment gave relief. Progressive emaciation and loss of strength, with indigestion, followed. Not until within two months of his death was the diagnosis of cancer made out. At this time there was a small tumor in the region of the pylorus, yet there was but little vomiting. Death took place in March of 1872. The autopsy revealed a scirrhus deposit along the greater curvature of the stomach.

The liver was healthy, except one or two small nodules in the left lobe. The gall bladder was distended with bile. The head of the pancreas was covered with a scirrhus tumor measuring three inches in diameter, pressing upon the ductus communicus cholidochus and obliterating the pancreatic duct. It was the opinion of my professional brethren present that the pancreatic tumor was the primary deposit, being more extensive, etc., than the others.

Case 2.—Mr. O. M——, aged about 50, married, by occupation a farmer. Had been an invalid several years; had dyspepsia; was relieved by tonics and pepsin; was not able to digest fatty food of any kind. I was called to see him in the Fall of 1875; found him suffering pain in the region of the pylorus, bright yellowness of conjunctiva and skin; pulse not accelerated. Complained of giddiness and some nausea. Diagnosed jaundice from obstruction. Treatment gave little relief; stools remained clay-colored until the day he died. Emaciation progressed rapidly, though food was taken in large quantities. Fatty dejections and very fetid. Patient died in March, 1876. Some twelve hours before death diarrhœa came on; after suffering severe pain he had two stools of the color of bile, but darker. The dejections were not fetid. The *post mortem* examination revealed a tumor over three inches in diameter covering the head of the pancreas and obliterating the pancreatic and bile ducts. The tumor had adhered to the duodenum and covered more than half its circumference for a length of two and one-half inches. A channel or duct, large enough to admit the index finger, had ulcerated from the dilated bile duct above through the tumor to the duodenum below. Through this the bile had undoubtedly discharged before death as the gall bladder, though very much dilated, was not distended. The stomach was healthy. The liver seemed comparatively healthy, except one or two whitish-yellow patches (very small). We were unable to determine their character, though presumably the same as the primary tumor, which was well marked scirrhus.

Case 3.—Mrs. C——, sister of case 1st, aged 48, married. Clinical history about the same as case 1st; cancerous cachexia well marked. About two months before death persistent vomiting set in. Autopsy revealed the same cancerous growth surrounding the head of pancreas, but not obliterating the choleric duct; also scirrhus of the pylorus very nearly closing the orifice.

CASE 4.—Mr, W——, aged 60, married; by occupation a farmer. Was called to see him in March, 1878; found pulse sixty per minute, cold extremities, yellowness of conjunctiva, stools were clay-colored and very offensive; appetite gone and bowels constipated. I diagnosed jaundice from obstruction, and treated him accordingly, without effect except to improve the appetite and digestion. In June I gave my diagnosis as "the obstruction is probably cancerous," though there was as yet but little acute pain, the patient not speaking of it unless asked. The stools were throughout of a clay color, as they had been for two months previous to my visiting him. His death occurred in August, 1878. The autopsy revealed cancerous deposit throughout the liver, though in small nodes, except near the outer border of the right lobe. The liver weighed seven pounds and two ounces. The gall bladder was very much distended with bile. The duct was dilated to near the junction with the pancreatic duct, so that when flattened it measured one inch and a half in width. The head of the pancreas was covered by a mass, evidently scirrhus in character, three inches in diameter and involving also the duodenum. The symptoms, common to the foregoing case: first, pain in the epigastric region, extending downward toward the umbilicus. Second, progressive emaciation. Third, the presence of a tumor, though this was often difficult to distinguish with certainty. Fourth, on pressure the pulsation of the aorta was plainly felt, being communicated through the tumor to the fungus. Fifth, only occasional acceleration of the pulse. Sixth, cancerous cachexia becomes well marked toward the close of life; also, at this time the character of the pain becomes more diagnostic. But, notwithstanding this, the diagnosis was not easily made out. It was not difficult to find physicians who would encourage a cure even within a month or two of the patient's death.

Now the query: Is not scirrhus of the pancreas a more frequent cause of death than is generally supposed? If it is so, any symptoms which may make the diagnosis more easy and certain, will be of value, as it will prevent patients from being subjected to a worse than useless treatment because of a wrong diagnosis.

